# Topical diclofenac epolamine patch 1.3% for treatment of acute pain caused by soft tissue injury

**DOI:** 10.1111/j.1742-1241.2010.02474.x

**Published:** 2010-10

**Authors:** B H McCarberg, C E Argoff

**Affiliations:** 1 Kaiser Permanente Health Care, Chronic Pain Management ProgramEscondido, CA, USA; 2 Comprehensive Pain Program, Department of Neurology, Albany Medical CenterAlbany, NY, USA

## Abstract

Acute pain caused by musculoskeletal disorders is very common and has a significant negative impact on quality-of-life and societal costs. Many types of acute pain have been managed with traditional oral non-steroidal anti-inflammatory drugs (NSAIDs) and selective cyclooxygenase-2 inhibitors (coxibs). Data from prospective, randomised controlled clinical trials and postmarketing surveillance indicate that use of oral traditional NSAIDs and coxibs is associated with an elevated risk of developing gastrointestinal, renovascular and/or cardiovascular adverse events (AEs). Increasing awareness of the AEs associated with NSAID therapy, including coxibs, has led many physicians and patients to reconsider use of these drugs and look for alternative treatment options. Treatment with NSAIDs via the topical route of administration has been shown to provide clinically effective analgesia at the site of application while minimising systemic absorption. The anti-inflammatory and analgesic potency of the traditional oral NSAID diclofenac, along with its physicochemical properties, makes it well suited for topical delivery. Several topical formulations of diclofenac have been developed. A topical patch containing diclofenac epolamine 1.3% (DETP, FLECTOR® Patch), approved for use in Europe in 1993, has recently been approved for use in the United States and is indicated for the treatment of acute pain caused by minor strains, sprains and contusions. In this article, we review the available clinical trial data for this product in the treatment of pain caused by soft tissue injury.

**Review Criteria**

Information was gathered through a search of MEDLINE, Derwent Drug File, BIOSIS and EMBASE databases on diclofenac epolamine topical patch and diclofenac hydroxyethylpyrrolidine patch, for publications from 1985 to present, in any language. Additional sources used in the development of this article include product prescribing information and relevant conference poster presentations.

**Message for the Clinic**

Interest in topical NSAIDs that provide analgesia while minimising systemic absorption has increased as a result of growing awareness of adverse events associated with systemic therapy. The diclofenac epolamine topical patch 1.3% (DETP), available in Europe since 1993, was recently approved in the United States for topical treatment of acute pain caused by minor strains, sprains and contusions. Newly available postmarketing surveillance data covering approximately 14 years strengthen available safety data. DETP continues to offer a viable treatment option in patients with acute pain caused by minor strains, sprains and contusions.

## Introduction

In the United States, between 2004 and 2005, soft tissue injuries such as strains, sprains and contusions each accounted for approximately 18% of initial visits to the emergency department for injuries (1). The most common sports-related musculoskeletal injury is ankle sprain (2,3), for which approximately 2 million people seek medical treatment each year (3). Other common soft tissue injuries occur in the elbow (4) and knee (5).

Use of an analgesic medication, in particular the judicious use of oral traditional non-steroidal anti-inflammatory drugs (NSAIDs) and cyclooxygenase-2 inhibitors (coxibs), has been shown to be beneficial in reducing pain and swelling in acute soft tissue injuries (6,7). NSAID treatment is included in current guidelines for the treatment of acute ankle sprain, which focuses on the reduction of inflammation and pain following injury (8,9). Interest in topical NSAIDs for this use has been increasing as a result of growing awareness of the adverse effects (AEs) associated with systemic NSAIDs and coxibs. Three topical NSAID formulations, all salts of diclofenac, are approved for use for pain indications in the United States: diclofenac epolamine topical patch 1.3% (DETP; FLECTOR® Patch) (10); diclofenac sodium gel 1% (Voltaren® Gel) (11) and diclofenac sodium topical solution 1.5% (Pennsaid®) (12).

### NSAIDs overview

The consequences associated with pain include negative effects on quality-of-life and societal costs (13). Musculoskeletal pain is a common problem often treated with NSAIDs and coxibs. Postmarketing AE monitoring of the use of oral NSAIDs and coxibs has brought the issue of safety to the forefront (13,14). The benefits of oral NSAID therapy must be weighed against its potential for serious side effects including cardiovascular events (14), gastrointestinal (GI) ulceration/bleed (15,16) and renal side effects (17). The potential for GI AEs is an especially important concern for elderly patients (15,18).

Use of oral NSAIDs has been associated with a significantly increased risk of GI complications (19); among patients in the primary care setting, the prevalence of NSAID-associated ulcers was found to be 16% (20). Although less clinically detrimental than ulcers and GI bleeds, dyspepsia is a far more prevalent complication of NSAID therapy, conferring a significant clinical burden (21). Oral NSAID use has been shown to increase the risk of dyspepsia by approximately 40% (22); because of this increased risk, GI co-medications such as proton pump inhibitors are often required (15). In addition, GI AEs, including nuisance symptoms such as dyspepsia, upper abdominal pain and general abdominal pain, are among the most common reasons for discontinuation of oral NSAID therapy (23).

The GI tolerability and favourable systemic toxicity profile observed with topical NSAIDs may be a result of low systemic blood concentrations (18,24). Heyneman et al. (25) reviewed both single- and multiple-dose NSAID absorption studies. After topical NSAID administration, studies showed that peak plasma levels of the NSAID moiety were less than 10% of those obtained after oral administration (25). The pharmacological action of topical NSAIDs is exerted at the local level and is not dependent on systemic absorption (18,24).

Topical agents that are now available include NSAIDs, counter-irritants (e.g. capsaicin) and local anaesthetics, such as the lidocaine patch 5% (24,26). A clear distinction must be made between incidental absorption from topically applied drugs and that of transdermally absorbed drugs, whose action depends on systemic absorption (e.g. fentanyl, nicotine patches) (18,24,27). Although both types of formulations are applied directly to the skin, transdermal formulations are specifically designed to facilitate drug diffusion through the various layers of the skin into the systemic circulation (28) with the goal of achieving systemic levels comparable with those obtained with oral medications (24). For a topical drug formulation, however, the site of activity is the tissue directly underlying the application site, including the soft tissue and peripheral nerves (24,29). A topical drug uses transcutaneous delivery to penetrate the stratum corneum and reach its site of action (18). Serum levels generally remain relatively low and, consequently, systemic side effects or drug–drug interactions are significantly less likely (24).

The goal of topical agents is to achieve similar efficacy to oral formulations with potentially lower systemic side effects (24). Penetration studies confirm that topical NSAIDs reach therapeutic concentrations underneath the site of application equivalent or greater to those seen with larger doses of oral NSAIDs (18,24,25). Another key factor in determining the effectiveness of topically applied NSAIDs is their intrinsic pharmacological potency in terms of cyclooxygenase (COX) inhibition. Those traditional NSAIDs with a high intrinsic potency include flurbiprofen, piroxicam and diclofenac (18,30). Salicylates have much lower potencies and therefore are much less likely to achieve therapeutic concentrations via the topical route (18).

### Efficacy of topical NSAID therapy

NSAIDs primarily inhibit the COX pathway responsible for transforming arachidonic acid to prostaglandins, prostacyclins and thromboxanes (7). A meta-analysis in 2004 by Mason et al. (31) showed topical NSAIDs to be effective and safe in treating acute painful conditions for 1 week. This systemic review of 26 double-blind, placebo-controlled trials showed clinically significant efficacy in 19 of 26 trials, with a pooled relative benefit of 1.6 and number needed to treat of 3.8 vs. placebo to achieve an outcome of approximately 50% reduction in pain at 7 days (31). Results were consistent regardless of end-point reported and condition treated (31). Three trials (*N* = 433) that compared topical vs. oral NSAIDs showed similar efficacy (31). Local AEs (4% topical, 5% placebo), systemic AEs (3% topical, 2% placebo) and withdrawals because of AEs (topical and placebo both 1%) were not statistically different from placebo (31). Vaile et al. (32) summarised double-blind trials of topical NSAIDs vs. topical placebo for soft tissue injuries. Topical NSAIDs that showed a significant benefit with regard to pain control included 2.5% niflumic acid gel (33) and ketorolac gel (34). Several topical agents, including ketoprofen 2.5% gel, indomethacin 1% spray and ibuprofen 5% gel, did not show significant benefit vs. placebo in pain or function; in many cases, however, trends in pain improvement favouring the active agent were evident (32).

### Safety of topical NSAIDs

Topical NSAIDs may have potential advantages when compared with oral NSAIDs. Several studies demonstrate that, perhaps because of low systemic concentrations, topical NSAIDs have a reduced risk of upper GI complications such as gastric and peptic ulcers, and GI nuisance symptoms such as dyspepsia (23,35,36), as well as a lack of drug–drug interactions (29), which leads to minimal side effects in general. However, although AE incidence is low, larger controlled, head-to-head comparisons of topical and oral NSAIDs should be conducted to confirm any safety benefit. Dose titration is often not needed with topical NSAIDs, thus reducing the time to effective pain control (29). In addition, the ease of use of a topical NSAID, as well as the subjective benefit associated with applying a topical preparation to a painful site, may result in better acceptance by patients and a possible increase in compliance (18). When given a choice, many elderly patients with mild, transient knee pain tend to choose topical NSAIDs over oral NSAIDs (37–39). An exploratory analysis of factors that influence patient choice of therapy found risk of AEs, level and extent of pain, clinician’s advice and convenience to contribute to patient preferences; patients with transient pain were more likely to prefer topical formulations for the short-term management of pain (39).

Topical NSAIDs have been licenced for nearly 30 years in Europe, Japan and South Africa (25). A large variety of topical NSAID formulations are available (24,32), ranging from ointments and creams to gels and patches (Table 1). Topical NSAIDs have now been introduced in the United States. Ongoing and recently completed clinical trials of topical NSAIDs in the United States include topical diclofenac for the treatment of knee osteoarthritis (phase 3; Nuvo Research Inc., Mississauga, ON, Canada) and ketoprofen topical patch (20%) for the treatment of pain associated with osteoarthritis flare of the knee (phase 3; Endo Pharmaceuticals, Chadds Ford, PA, USA) (40,41).

**Table 1:** Topical NSAID formulations available worldwide (24,32)

Active ingredient	Formulation(s)
Diclofenac	Patch*, gel*, drops*
Indomethacin	Ointment, spray, gel
Ibuprofen	Cream, gel
Benzydamine	Cream
Salicylic acid	Cream, gel
Flurbiprofen	Patch, drops
Piroxicam	Gel
Felbinac	Gel, foam
Eltenac	Gel
Ketoprofen	Gel, foam
Ketorolac	Drops
Suprofen	Drops

*Formulation of diclofenac approved in the United States. NSAID, non-steroidal anti-inflammatory drug.

### The diclofenac epolamine topical patch 1.3%

One of the topical NSAID formulations approved in the United States is the DETP. In contrast to other conventional formulations (e.g. creams, gels), DETP provides a defined dose to a defined area of skin for 12 h, requiring twice per day application (10). DETP has recently been approved for use in the United States for the topical treatment of acute pain caused by minor strains, sprains and contusions (10).

### Compound characteristics

DETP, marketed as FLECTOR® Patch (King Pharmaceuticals®, Inc., Bridgewater, NJ, USA), developed and patented by IBSA Institut Biochimique SA (Lugano, Switzerland) (42) is the first NSAID topical patch available in the United States. Diclofenac epolamine is also known as diclofenac-*N*-(2-hydroxyethyl)-pyrrolidine (DHEP) (10,43). The diclofenac molecule, in its acidic form, is hydrophobic with very low solubility in water (43). Studies have shown that the epolamine salt of diclofenac has greater solubility in water and non-polar solvents (1-octanol) than other diclofenac salts that have been studied (43,44). High concentrations of aqueous diclofenac epolamine solutions exhibit surfactant behaviour (43). The solubility and surfactant properties of diclofenac epolamine enhance its membrane permeability (43,44).

### Mechanism of action

**Figure 1:**
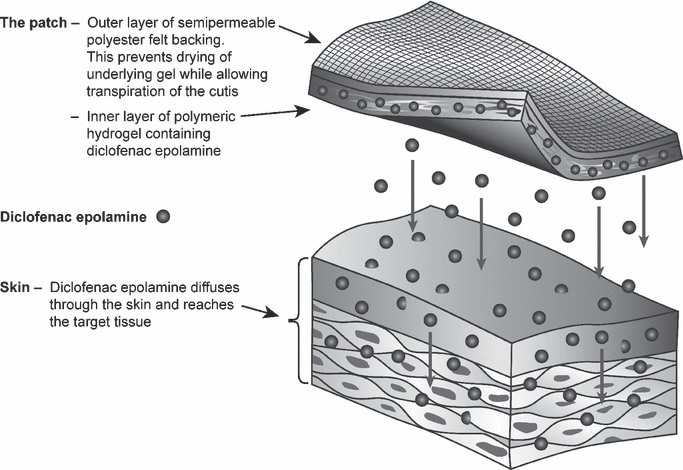
Diagrammatic representation of the diclofenac epolamine topical patch at the site of application

DETP is a ready-to-use adhesive patch composed of two layers: an outer layer of non-woven polyester felt backing and an adhesive inner layer containing 1.3% of diclofenac epolamine in a polymeric hydrogel (Figure 1) (10). The entire patch is covered with a polypropylene film-release liner that is removed prior to application of the patch to the skin (10). The felt backing prevents the hydrogel from drying out and also hydrates the covered area of skin during perspiration to aid in the absorption of active ingredient. Whether the patch is used at rest or during moderate exercise, there are no clinically relevant differences in diclofenac plasma concentrations (10). When applied to intact skin, the hydrogel enables gradual and sustained release of the active agent into the skin to provide local analgesia over a 12-h period (10).

The anti-inflammatory, analgesic and antipyretic actions of diclofenac are well established (45,46). It is the most widely prescribed, traditional NSAID with more than 30 years of clinical use (42,47). The characterisation of diclofenac as a traditional NSAID, inhibiting both COX-1 and COX-2, was further confirmed in a recent randomised controlled clinical study comparing diclofenac with etoricoxib and celecoxib in healthy subjects (48). Sodium or potassium salts of diclofenac are used in preparations for oral administration and the diethylammonium and sodium salt have been used in topical gel form (42,49). Penetration of diclofenac into the muscular tissue underlying the patch after application of DETP has been indirectly demonstrated by the resulting significant increase of the muscular pain threshold (35).

### Pharmacokinetics and metabolism

#### Absorption

Low systemic absorption of diclofenac following DETP application has been shown in a study by Rusca et al. (50). Over 12 h, the steady state plasma concentrations of diclofenac following DETP application (twice daily for four consecutive days) were < 1% of those following a single 50-mg oral dose of diclofenac sodium (50). Based on two additional studies assessing the pharmacokinetics of diclofenac potassium over 6 h, the relative bioavailability of DETP was estimated to be 0.9–1.7% of 75 mg diclofenac potassium (51–53).

Other studies show that following a single application of DETP on the upper area of the inner arm, peak plasma concentrations of diclofenac (range: 0.7–6 ng/ml) were noted between 10 and 20 h after application (10). Plasma concentrations of diclofenac in the range of 1.3–8.8 ng/ml were noted after 5 days with twice-daily DETP application (10). Gallacchi et al. (54) assessed blood and synovial levels of diclofenac after repeated application of DETP twice daily for four consecutive days in patients with joint effusion (*N* = 8). Synovial fluid concentrations of diclofenac were 36% of concentrations found in plasma. These concentrations indicate direct transport of diclofenac across the skin to reach the synovial fluid compartment. The mean plasma concentration was 3.62 ng/ml at 4 h after the last application (54). Steady state plasma diclofenac concentrations evaluated in healthy subjects in three studies between 1998 and 2002 were achieved before day 3 and were approximately 3 ng/ml (51). During 12 h of DETP application, cycling for 20 min/h did not affect the pharmacokinetic profile of diclofenac (51). Gschwend et al. (2005) (55) assessed blood concentrations of diclofenac after twice-daily application of DETP for four consecutive days in healthy volunteers (*N* = 24). The maximum peak plasma concentrations were 1.55 ng/ml (0–12 h) and 1.57 ng/ml (0–24 h) (55). These results can be compared with studies of oral diclofenac sodium reviewed by Brogden et al. (1980) (46). In a study using an oral diclofenac sodium single dose of 25 mg, peak plasma concentrations of 720–1100 ng/ml were reported in Geiger et al. (1975) (56). In another study, an oral diclofenac sodium single dose of 50 mg was used in fasting young (age < 22 years) and older (age > 62 years) women; mean peak plasma concentrations in this study were 1500–1600 ng/ml (Willis and Kendall 1978) (57).

#### Distribution

Diclofenac is highly bound to serum proteins (46) and has a very high affinity (> 99%) for human serum albumin (10). A low volume of distribution allows for preferential distribution to the site of inflammation and persistent concentrations in the synovial fluid, often with greater concentrations than those found in plasma (58,59).

#### Metabolism and excretion

The plasma elimination half-life of diclofenac after application of DETP is approximately 12 h (10). Diclofenac is eliminated through metabolism and subsequent urinary and biliary excretion of the glucuronide and the sulphate conjugates of the metabolites (10).

### Clinical efficacy

#### Soft tissue injuries

The efficacy of DETP has been demonstrated in a number of studies for the treatment of strains and sprains. Jenoure et al. (60) performed an open-label study of 101 patients with minor sports injuries. Overall, treatment was associated with a 61% reduction in pain on pressure and a 60% reduction in spontaneous pain [verbal and visual analog scale (VAS)] after 2 weeks of DETP treatment (60). Analgesic effects were apparent on day 7, with a mean 28% reduction of spontaneous pain (60). As judged by the investigators, all but 18 patients (17.8%) received clinical benefit treatment. Global assessments for tolerability of DETP, expressed by the investigator and patients, were ‘good’ or ‘excellent’ in all patients studied (60). A multicenter, double-blind, placebo-controlled study (*N* = 140) demonstrated the effectiveness and tolerability of DETP vs. placebo in reducing the acute pain of ankle sprain (61). A significant reduction in spontaneous pain was observed for DETP vs. placebo starting at 3 h (p = 0.005) and persisting until days 3 (p = 0.004) and 7 (p = 0.0008). DETP was assessed as being superior to placebo by both patients and physicians and showed favourable local tolerability compared with placebo. Joussellin (62) conducted a confirmatory, multicenter, randomised, placebo-controlled, parallel-group study in patients with acute ankle sprain using comparable methodology. Patients (*N* = 134) who sustained an ankle sprain < 48 h before study entry and with spontaneous pain of at least 50 mm on a 1- to 100-mm VAS were treated with DETP, applied every morning for the 7-day study period (62). DETP showed significantly greater efficacy in the primary criteria, pain on movement, when compared with placebo from the fourth hour following the first application until the end of the study (62). All secondary criteria (pain at rest, pain on passive stretch, pain on pressure, pain while leaning on single foot) were significantly improved from day 3 in the DETP group compared with the placebo group (62). Global judgment of efficacy by both patients and investigators confirmed the superior efficacy of DETP vs. placebo (62). No adverse events (AEs) were observed. Joussellin concluded that the anti-inflammatory and analgesic properties of DETP, combined with its local tolerability and ease of use, make it a therapeutically useful treatment option for minor ankle sprains (62). Rowbotham et al. (63) evaluated the efficacy and safety of DETP in the treatment of minor sports injuries. Compared with placebo, time to pain resolution [8.8 days (95% Confidence Interval, CI: 7.5–10.3) vs. 12.4 days (CI: 10.3, > 15 days)] and reduction in pain scores (starting day 6 and continuing to day 13, p = 0.042) were significantly better after administration of DETP (q12 h) in subjects with acute pain caused by minor sports injuries (63).

Galer et al. (36) studied the efficacy and tolerability of DETP vs. placebo for the treatment of sports-related, soft tissue injuries (strains, sprains or contusions) in a multicenter, randomised, parallel-group, 2-week study (*N* = 222). DETP achieved statistically significant pain relief vs. placebo as measured by summed pain intensity difference at days 3 (p = 0.036) and 14 (p = 0.048) (36). As measured by daily diaries, total pain relief scores and patient-rated functional improvement scores were significantly higher with DETP compared with placebo at days 7 and 14 (p ≤ 0.037). There was no difference in pain on pressure between groups. A similar incidence of AEs was observed in both treatment groups (36).

#### Other conditions

Although DETP is indicated in the United States for the treatment of acute pain caused by minor strains, sprains and contusions, in other countries it has been used for treatment of pain caused by other conditions including osteoarthritis (10,64–66). Osteoarthritis Research Society International 2008 recommends use of topical NSAIDs for treatment of knee osteoarthritis (67). Jenoure et al. (68) studied the efficacy of DETP in epicondylitis (tendinopathic pathology) for a 2-week period with a 2-week posttreatment follow-up. This was a multicenter, double-blind, randomised, placebo-controlled study in 85 patients. DETP was significantly superior to placebo in reducing spontaneous pain measured using a 5-point verbal scale. At day 28, the percentage of patients experiencing moderate-to-severe pain was 17.9% in the DETP group vs. 47.3% in the placebo group (p < 0.01) (68).

Rosenthal et al. (69) compared DETP, applied twice daily, with diclofenac diethylammonium (DDA) emulgel, applied four times daily, in patients (*N* = 190) with localised inflammatory diseases. In this controlled, randomised, 2-week study both treatments decreased pain; however, significantly more patients and investigators reported superior efficacy with DETP than with DDA (p < 0.001). The authors theorised that better results were because of the constant release of active substance by DETP vs. the DDA emulgel, as the latter required four applications daily. Both treatments were well tolerated (69).

#### Safety and tolerability

The most common AEs reported with DETP are mild local skin reactions; topical NSAIDs, including DETP, should not be applied to skin with lesions or dermatologic conditions. In addition, topical NSAIDs, like all NSAIDs, have a black box warning for increased risk of cardiovascular events and serious GI AEs (10). Pooled safety data were evaluated from 17 clinical trials with a total of 1344 patients receiving 22,949 patches (average exposure 15.3 patches per patient) (70,71). The tolerability of DETP was evaluated using the incidence of skin irritation phenomena, sensitisation, phototoxicity and/or photoallergy (70). Of the 1344 patients, only 43 (3.1%) had several moderate cutaneous reactions consisting of erythema, pruritus or petechiae; no serious local or systemic manifestations were observed (70,71). Across clinical trials, the most common AEs were skin reactions at the site of treatment and were similar for DETP and placebo. These included (DETP vs. placebo): pruritus (5% vs. 8%); dermatitis (2% vs. < 1%) and burning (< 1% vs. 1%). Only 3% of patients in both the DETP and placebo patch groups discontinued treatment as a result of an AE (10). In an analysis of data from 1997 to 1999 on the use of DETP twice daily for a minimum of 14 days in children aged 8–15 years with minor sports injuries, there were no reported AEs (72).

Approximately 175,000,000 patches have been dispensed in over 40 countries around the world, representing over six million unique patient exposures. Based on postmarketing reports from Europe and the United States, covering June 1993 to January 2008, 178 AEs have been reported in 108 patients, most commonly related to application site reactions (71).

## Conclusions

Increasing awareness of the AEs associated with oral traditional NSAID therapy and coxibs has led many physicians and patients to reconsider use of these drugs despite their efficacy for the management of mild-to-moderate pain. One alternative has been to develop ways to improve the safety of NSAIDs without diminishing their efficacy. These efforts include the introduction of topical NSAID formulations, which have been used as clinically effective analgesic agents in Europe and throughout the world for almost 30 years and are now approved for use in the United States.

In contrast to conventional formulations, such as creams, gels and sprays, DETP provides a defined dose to a defined area for an extended period of time (typically 12 h), as opposed to topical NSAID gels or creams that are applied up to four times daily (11,12). Application of a cream or gel is at the discretion and capability of the patient, and the dosage may depend to some extent on diligence in following treatment instructions. In addition, application of the patch is devoid of the messiness or staining of the skin that may occur while applying creams or gels. Both physician and patient global assessment data from clinical trials were very favourable for DETP (60–62,68,69).

Topical NSAID formulations may be important for patients who are at risk for GI AEs that may result from the use of oral NSAIDs, because topical NSAIDs expose patients to lower systemic levels of diclofenac. Risk factors for serious GI events include older age, prednisone use, previous NSAID GI side effects and prior GI hospitalisation (73). Some patients, even those without identifiable risk factors, may be familiar with the risks associated with oral NSAIDs and may simply prefer the low systemic exposure associated with DETP use. In addition, although less serious, GI AEs such as dyspepsia are among the most prevalent AEs leading to discontinuation of oral NSAID therapy. Reducing these nuisance AEs can lead to increased tolerability, compliance and patient satisfaction.

Many clinicians and patients are familiar with patch formulations. For example, the lidocaine patch has demonstrated efficacy in the treatment of neuropathic pain types, mainly by stabilising neuronal activity, without significant side effects (26). Topical patches offer a local route of administration for NSAIDs, which have both analgesic and anti-inflammatory properties. The different indications for these patches are reflective of the potential differences in mechanisms of action. In addition, other formulations, such as liquids, foams, gels and creams, are likely to offer advantages for patients with pain in areas not amenable to patch application.

Over the next few years, we will likely witness an effort dedicated towards optimising delivery of NSAIDs while minimising associated risks, along with an increased acceptance of topical formulations. It is likely that additional topical analgesic formulations will be introduced, and more clinical studies will be performed both in support of these applications and in light of the new evidence about the risks of oral NSAIDs.

Placebo-controlled studies included in the present report found that patients treated with DETP experienced a significantly faster and greater improvement in pain compared with patients treated with placebo. Taking into consideration the safety profile of topical diclofenac formulations in general, and diclofenac epolamine in particular, as well as the low incidence of local adverse reactions and lack of systemic side effects, DETP offers a viable treatment option in patients presenting with acute pain caused by minor strains, sprains and contusions.
